# Advances on the roles of tenascin-C in cancer

**DOI:** 10.1242/jcs.260244

**Published:** 2022-09-14

**Authors:** Alev Yilmaz, Thomas Loustau, Nathalie Salomé, Suchithra Poilil Surendran, Chengbei Li, Richard P. Tucker, Valerio Izzi, Rijuta Lamba, Manuel Koch, Gertraud Orend

**Affiliations:** ^1^ The Tumor Microenvironment Laboratory, INSERM U1109, Hôpital Civil, Institut d'Hématologie et d'Immunologie, 1 Place de l'Hôpital, 67091 Strasbourg, France; ^2^ Université Strasbourg, 67000 Strasbourg, France; ^3^ Fédération de Médecine Translationnelle de Strasbourg (FMTS), 67000 Strasbourg, France; ^4^ Department of Cell Biology and Human Anatomy, University of California at Davis, 95616 Davis, CA, USA; ^5^ Faculty of Biochemistry and Molecular Medicine, University of Oulu, FI-90014 Oulu, Finland; ^6^ Faculty of Medicine, University of Oulu, FI-90014 Oulu, Finland; ^7^ Institute for Dental Research and Oral Musculoskeletal Research, Center for Biochemistry, Center for Molecular Medicine Cologne (CMMC), Faculty of Medicine and University Hospital Cologne, University of Cologne, Joseph-Stelzmann-Str. 52, 50931 Cologne, Germany

**Keywords:** Immunity, Metastasis, Tenascin-C, Tumor

## Abstract

The roles of the extracellular matrix molecule tenascin-C (TNC) in health and disease have been extensively reviewed since its discovery over 40 years ago. Here, we will describe recent insights into the roles of TNC in tumorigenesis, angiogenesis, immunity and metastasis. In addition to high levels of expression in tumors, and during chronic inflammation, and bacterial and viral infection, TNC is also expressed in lymphoid organs. This supports potential roles for TNC in immunity control. Advances using murine models with engineered TNC levels were instrumental in the discovery of important functions of TNC as a danger-associated molecular pattern (DAMP) molecule in tissue repair and revealed multiple TNC actions in tumor progression. TNC acts through distinct mechanisms on many different cell types with immune cells coming into focus as important targets of TNC in cancer. We will describe how this knowledge could be exploited for cancer disease management, in particular for immune (checkpoint) therapies.

## Teaser

**Summary:** A review of recent insights into the roles of the extracellular matrix molecule tenascin-C in tumorigenesis, tumor immunity and metastasis, and how this could be exploited for cancer disease management.

## Introduction

Some gene mutations are known to lead to tumor formation; however, the extracellular matrix (ECM) and the 3D context matters for these mutations to instigate a tumor (reviewed in Bissell and Hines, 2011). The glycoprotein tenascin-C (TNC) is an ECM molecule that plays multiple and context-dependent roles in cancer that have been comprehensively reviewed elsewhere (Midwood and Orend, 2009; Midwood et al., 2016; Lowy and Oskarsson, 2015; Tucker and Degen, 2022). Here, we will summarize and critically review new information on how TNC impacts tumor progression and will put forward new concepts of how TNC regulates tumor immunity, which offers potential novel targeting opportunities.

**Fig. 1.**
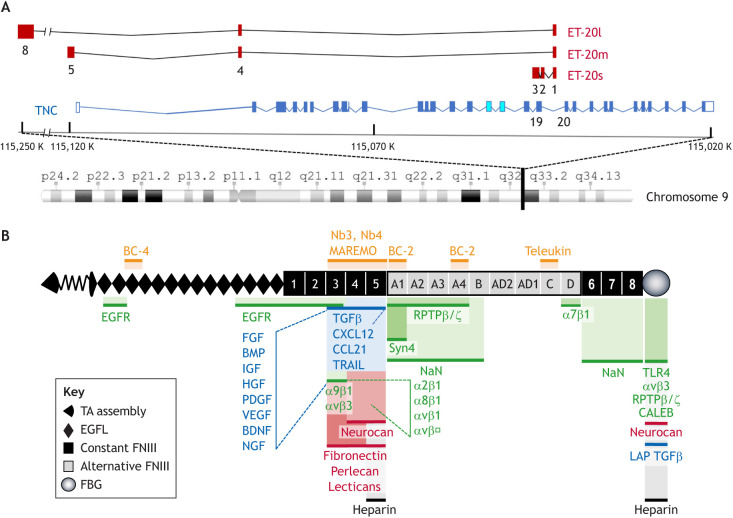
**Chromosomal locus encoding TNC and ET-20, and domain structure and interaction partners of TNC.** (A) Chromosomal location of the TNC protein-encoding sequence and sequence for the ET-20 lncRNA. The human *TNC* gene is shown schematically, with coding exons as dark blue boxes and non-coding exons as white boxes outlined with blue. The two exons in light blue encode the variable FNIII domains AD2 and AD1; these are not part of ENST00000350763 but have been added here. The *TNC* gene is found on the reverse strand of Chromosome 9 between 115,120 K and 115,020 K. Variants of the lncRNA ET-20 are found on the forward strand. Sequences homologous to the first exon of the three murine ET-20 variants are found between TNC exons 19 (encoding FNIII D) and 20 (encoding part of FNIII 6). Similarly, homologous sequences for the remainder of the short ET-20s sequence partially overlap with TNC exon 19. The positions of the exons encoding the middle-sized ET-20m and long ET-20l are mapped here based on their locations in the mouse genome. ET-20l exon 5 is predicted to be found near 115,250 K. ET-20l exons 5–7 are present in the region that is depicted as a gap. (B) Cartoon of the human TNC protein. Interaction partners include ECM molecules (red), cell surface receptors (green), soluble molecules (blue) and others (black). Therapeutic molecules with known interaction sites, such as antibodies [BC-2, BC-4 and Teleukin (also known as F16)], nanobodies Nb3, Nb4 and MAREMO peptides are indicated (orange). Note, that the TN3–TN5 and FBG domains are hotspots for molecular interactions. In addition to TGFβ, CXCL12, CCL21 and TRAIL, other soluble factors also bind to the same domains (TN4 and/or TN5), and several integrins bind to the TN3 domain. Other molecules such as annexin II, contactin, CCN2, collagen V, fibrillin-2, von Willenbrand factor (vWF), periostin, MMP2 and MMP3, as well as streptococcus and HIV have also been found to bind TNC, but without information on where exactly.

**Fig. 2.**
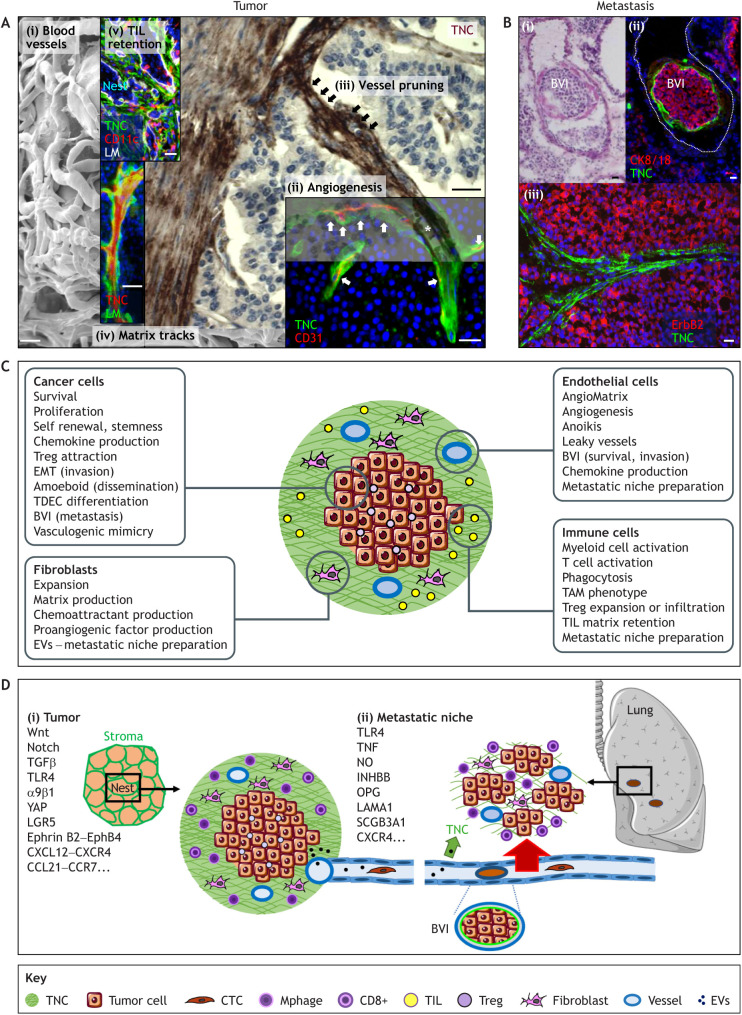
**Illustration of TNC expression and function in tumors and lung metastasis.** (A) Composite representation of TNC expression and function in the primary tumor and lung metastasis. Shown here are images derived from different tissues that have been assembled to provide a comprehensive visual display of the different sites of TNC expression. (i) Scanning electron micrograph (SEM) image of a PNET model tumor overexpressing TNC showing irregular and dysfunctional blood vessels with a small lumen diameter. Reproduced from Saupe et al. (2013), where it was published under a CC BY-NC-ND 3.0 license, under rights retained by G.O. (ii–v) Images of primary tumors with tissue staining for TNC in either green (immunofluorescence staining; v), red (immunofluorescence staining; iv) or brown [immunohistochemical (IHC) staining; iii]. (ii) Endothelial cells (CD31, red) are surrounded by freshly deposited TNC (arrows), reminiscent of pro-angiogenic TNC properties. Shown here is a human colorectal carcinoma. Reproduced from Spenlé et al. (2015a), where it was published under a CC-BY-NC-3.0 license, under rights retained by G.O. (iii) Vessel pruning. Continuum of an intact blood vessel (red blood cells inside lumen, asterisk) and pruned blood vessel (arrows) as determined by IHC indicating anti-angiogenic TNC properties. Shown here is a human insulinoma. Reproduced from Spenlé et al. (2015a), where it was published under a CC-BY-NC-3.0 license, under rights retained by G.O. (iv) TNC is deposited in several parallel aligned matrix fibers that generate matrix tracks (brown). The insert shows an enlarged image of such a track stained for laminin (LM, green) flanking TNC (red). Shown here is a murine PNET tumor. Reproduced from Spenlé et al. (2015a), where it was published under a CC-BY-NC-3.0 license, under rights retained by G.O. (v) Matrix tracks stained for TNC (green) and LM (white) separate tumor nests (DAPI, blue) and retained leukocytes (CD11c, red) inside the stroma. Shown here is a 4NQO-induced tongue OSCC. Reproduced from Spenlé et al. (2020) under rights retained by G.O. Fig. 2A is not published under the terms of the CC-BY license of this article. For permission to reuse, please see individual references. (B) TNC expression in lung metastasis. (i,ii) Pulmonary blood vessel with BVI as determined by either H&E (i) or staining for TNC (green) (ii). Clusters of CTC (CK8/18, red) are surrounded by TNC (green) and an outer endothelial monolayer. (iii) TNC expression in fibrillar matrix inside a pulmonary metastasis is reminiscent of matrix tracks seen in the primary tumor. Shown here is a MMTV-NeuNT tumor. Reproduced from Sun et al. (2019), where it was published under a CC-BY 4.0 license. Scale bars: 20 µm. (C) Regulation of cell phenotypes by TNC. Many tumors are compartmentalized into tumor cell nests that are demarcated by the surrounding stroma, which is inherently rich in TNC (green). TNC regulates the phenotypes of tumor cells, fibroblasts, ECs and immune cells as indicated, altogether orchestrating an immunosuppressive tumor microenvironment and promoting metastasis. BVI, blood vessel invasion; EMT, epithelial to mesenchymal transition; EV, extracellular vesicle; TAM, tumor-associated macrophage; TDEC, tumor cell-derived EC; TIL, tumor infiltrating leukocyte; Treg, T regulatory cells. (D) Effects of TNC-regulated signaling on the formation of local matrix niches in the primary tumor and at the metastatic site. Some molecules and pathways, discussed in this and other reviews, have been described to be exploited by TNC in the tumor and the metastatic niche. (i) Tumor niche. Schematic illustration of a tumor–stroma unit. Although DCs and tumor cells are expressing TNC, fibroblasts are the major source of TNC. Tumor cells and fibroblasts also express soluble factors that bind to TNC, which attract and retain TILs (DC, macrophages and CD8T cells) in the stroma. TNC also promotes infiltration of Treg into the tumor nest. (ii) Metastatic niche. Tumor cells and fibroblasts secrete EVs that travel through the circulation, either depositing TNC or inducing its expression at the metastatic site in the lung or lymph node. In addition, CTCs can also leave the primary tumor upon EMT or via amoeboid migration and reach the metastatic site, potentially attracted by TNC and/or TNC-induced factors. TNC promotes cell survival in CTCs while they circulate in the blood stream. EMT and the EC layers from the BVI promote pulmonary tissue entry. Crosstalk between tumor cells, macrophages and EC is instrumental in formation of the metastatic niche.

The human *TNC* gene is located on chromosome 9q33 and comprises 30 exons. Long non-coding RNAs (lncRNA) are also encoded within and spanning the *TNC* locus, in the opposite direction (Fig. 1A). The TNC protein is composed of distinct structural domains, a tenascin assembly domain (TA), epidermal growth factor-like repeats (EGF-L), fibronectin (FN) type III domains (FNIII), and a fibrinogen-like globe (FBG) (Midwood et al., 2016) (Fig. 1B). *TNC* mRNA can be alternatively spliced, and the protein can be modified by cleavage, glycosylation and even citrullination, generating many TNC proteoforms (Midwood et al., 2016; Giblin and Midwood, 2015; Giblin et al., 2020; Song et al., 2021). The *TNC* nucleotide sequence is highly conserved during evolution (Midwood et al., 2016); however, TNC appears to be less conserved in human tissues where mutation rates are high, which is similar to what is found for other matrisomal molecules (the matrisome is the group of proteins that comprise the core ECM and ECM-associated molecules) (Box 1). Whereas mutation rates affecting amino acid exchanges in normal blood cells is low, this is much higher in some cancer tissues which may be relevant in context of anti-tumor immune surveillance (Box 1). TNC can interact with many molecules through distinct interactions with cell surface receptors [e.g. toll-like receptor 4 (TLR4) and integrins], matrix molecules (e.g. FN, collagens and lecticans), soluble factors (e.g. growth factors and chemokines), and many other molecules, thus regulating divergent cell type-specific and context-dependent responses (Midwood et al., 2016) (Fig. 1B). TNC expression is tightly controlled. However, in cancer, TNC is highly expressed around blood vessels, in immune suppressive matrix niches, at the invasive front and in metastases (illustrated in Fig. 2A,B). Studies with mice lacking the TNC protein have been instrumental in revealing that high TNC levels enhance pathological phenotypes, such as chronic inflammation (e.g. of the joints), fibrosis (e.g. in the kidney) and cancer (Midwood et al., 2016; Bhattacharyya et al., 2022; Marzeda and Midwood, 2018; Lowy and Oskarsson, 2015). It is noteworthy that the TNC knockout (KO) mouse has been generated several times (Table S1), as it was difficult to reconcile that ablation of the TNC protein had a rather mild phenotype. However, TNC-KO mice have multiple phenotypes, such as for example impaired tissue repair upon injury. They likely would not survive due to aggressive and other aberrant behaviors (reviewed in Chiquet-Ehrismann and Tucker, 2011).

**Box 1. TNC mutations in human tissue**

TNC mutations from all available mutation-calling pipelines in The Cancer Genome Atlas (TCGA) (upper part) and, GnomAD (https://gnomad.broadinstitute.org/) and ClinVar (https://www.ncbi.nlm.nih.gov/clinvar/) repositories comprising mostly sequence information from blood samples. All mutations not affecting the TNC protein sequence were removed and positions were matched with the canonical protein sequence and PFAM (protein family database; https://pfam.xfam.org/) domain coordinates. Amino acid exchanges are shown along a TNC monomer. In TNC, 1736 mutations were found across the whole pan-cancer cohort and 1479 mutations in the GnomAD cohort. In the cancer cohort, 76% of mutations are transitions and 24% are transversions. The specification of mutations per tumor is shown in the right-hand graph. In comparison with the matrisome, and normalized by gene length, TNC is in the upper range of mutated genes, ranking 163 out of 816. Mutations in position 1725 (domain C, amino acid exchange S to L, occurring in 2.02% of all cancer samples) and position 1309 (domain A3, amino acid exchange R to C, occurring in 1.38% of samples) are more frequent. In comparison to the mutations in the cancer cohort, mutations in the GnomAD cohort are less frequent. Whether the reported mutations represent a gain- or loss-of-function effect is unknown.
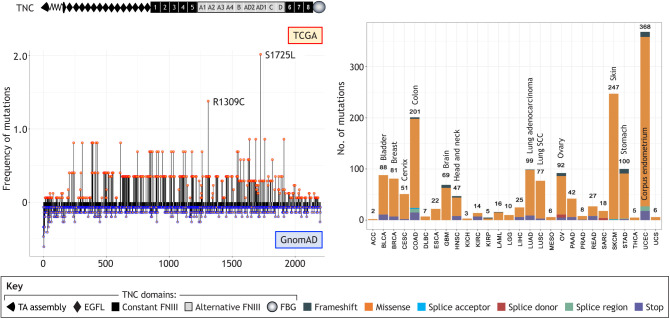

Abbreviations: ACC, adrenocortical carcinoma; BLCA, bladder urothelial carcinoma; BRCA, breast invasive carcinoma; CESC, cervical squamous cell carcinoma and endocervical adenocarcinoma; COAD, colon adenocarcinoma; DLBC, lymphoid neoplasm diffuse large B-cell lymphoma; ESCA, esophageal carcinoma; GBM, glioblastoma multiforme; HNSC, head and neck squamous cell carcinoma; KICH, kidney chromophobe; KIRC, kidney renal clear cell carcinoma; KIRP, kidney renal papillary cell carcinoma; LAML, acute myeloid leukemia; LGG, brain lower grade glioma; LIHC, liver hepatocellular carcinoma; LUAD, lung adenocarcinoma; LUSC, lung squamous cell carcinoma; MESO, mesothelioma; OV, ovarian serous cystadenocarcinoma; PAAD, pancreatic adenocarcinoma; PRAD, prostate adenocarcinoma; READ, rectum adenocarcinoma; SARC, sarcoma; SKCM, skin cutaneous melanoma; STAD, stomach adenocarcinoma; THCA, thyroid carcinoma; UCEC, uterine corpus endometrial carcinoma; UCS, uterine carcinosarcoma.

## Tenascin-C regulates tumor angiogenesis

TNC largely impacts vessel biology in different organs and context, such as by promoting vessel stability (preventing aneurism) (Imanaka-Yoshida and Matsumoto, 2018). TNC also affects tubular integrity in chronic kidney disease (Chen et al., 2019; Fu et al., 2017; Zhu et al., 2020). Formation of new blood vessels, which is termed angiogenesis, is considered as a hallmark of cancer (Hanahan and Weinberg, 2011), and here TNC plays multiple roles as illustrated in Fig. 2A. Connection to the vascular system is not only important for the delivery of nutrients and oxygen into the tumor but might also help leukocytes to reach the tumor, facilitating defense. Blood vessels also provide a route for tumor cell dissemination to form metastasis at distant sites, a major cause of patient death from cancer (Carmeliet and Jain, 2011). In a multistage tumorigenesis model with engineered TNC expression, a link between high TNC levels and an increased number of blood vessels that were, however, leaky was established (Saupe et al., 2013; Table 1). This likely mimics human glioblastomas (GBM) where a high amount of TNC (also around blood vessels) correlates with shorter patient survival (e.g. Herold-Mende et al., 2002). Gene expression analysis of angiogenic versus non-angiogenic dysplastic pancreatic islet tissue also revealed that TNC is a highly expressed member of the so-called AngioMatrix, a matrisomal signature that characterizes the angiogenic switch, which is associated with shortened cancer patient survival (Langlois et al., 2014, Table 1).

**Table 1.**
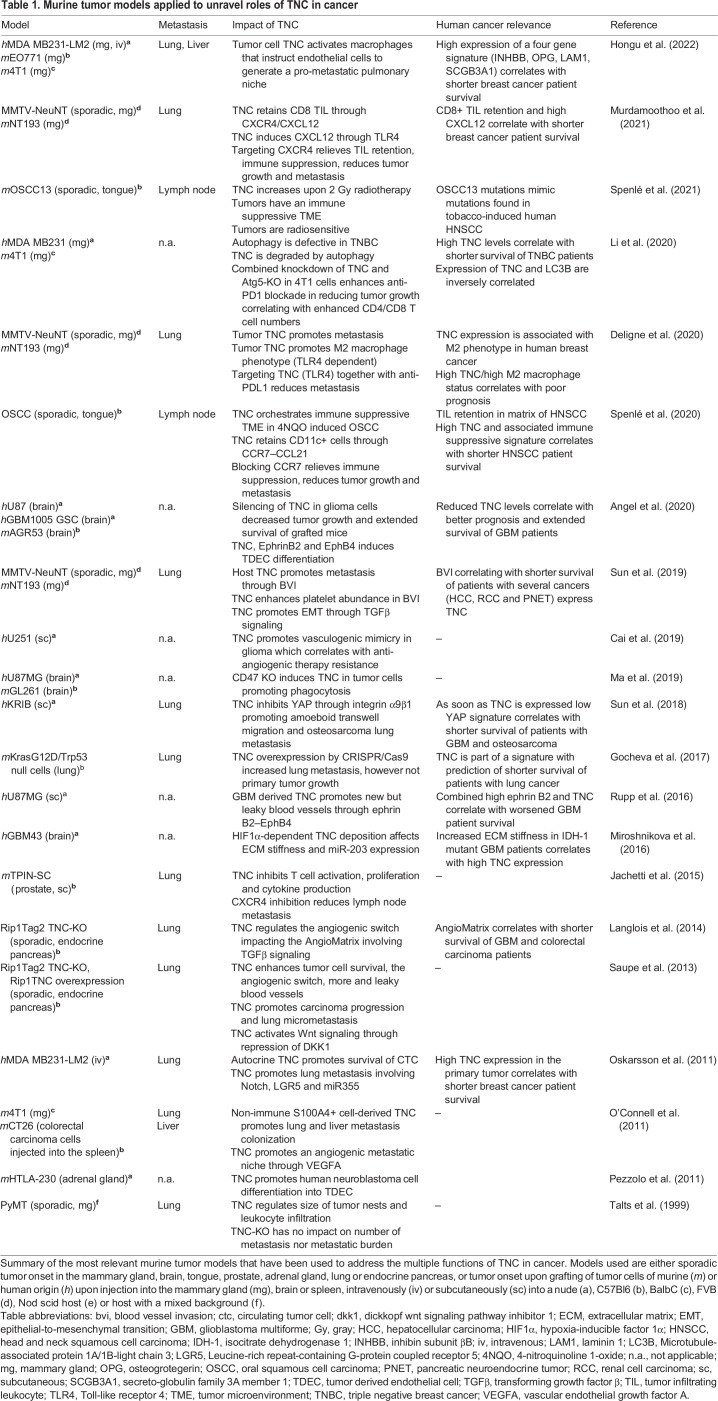
Murine tumor models applied to unravel roles of TNC in cancer

As TNC is anti-adhesive for most cells, a direct contact with TNC might explain cell death (anoikis) in anchorage-dependent endothelial cells (ECs) (Frisch and Francis, 1994). TNC-induced EC rounding has been correlated with an inactivation of the Hippo-pathway-associated molecule Yes-associated protein (YAP, also known as YAP1), which can act as a sensor of actin stress fibers (Cai et al., 2021). Accordingly, actin stress fibers were abolished upon cell detachment mediated by TNC, thus impairing nuclear shuttling of YAP to drive transcription of target genes (Rupp et al., 2016). Pro-survival YAP targets, including connective tissue growth factor (CTGF; also known as CCN2) and cysteine-rich angiogenic protein 61 (Cyr61; also known as CCN1), were downregulated (Rupp et al., 2016), raising the possibility that repression of pro-angiogenic factors plays a role in TNC-associated vessel leakiness. ECs were able to overcome TNC-induced cell death by activating Wnt signaling and subsequent induction of cellular FN to generate an insulating matrix layer (Radwanska et al., 2017).

In GBM, the tumor cells largely contribute to TNC expression (Huang et al., 2010). Gene expression and proteomic analysis of the secretome from cultured human glioblastoma cells with either high or engineered lowered TNC levels revealed that TNC induces several pro-angiogenic factors that promote EC expansion and tubulogenesis *in vitro*, and angiogenesis and tumor growth *in vivo* (Rupp et al., 2016). Inhibition of TNC-induced factor ephrin B2 signaling through its receptor EphB4 caused smaller and less vascularized tumors, phenocopying reduced angiogenesis in the TNC knockdown (TNC-KD) tumors (Table 1). Combined high expression of ephrin B2 and TNC was shown to be more powerful in predicting shorter patient survival than high expression of each factor alone. This did not only apply to GBM but also to lower grade glioma (LGG), thus opening new therapeutic opportunities (Rupp et al., 2016; Zhang et al., 2022). A role of TNC-induced ephrin B2 signaling was recently confirmed where, in addition to angiogenesis, the TNC–ephrin B2–EphB4 pathway also promoted tumor cell differentiation into ECs (TDECs), employing NF-κB signaling (Angel et al., 2020).

TNC also impacts ECs in several tumors with blood vessel invasions (BVIs), representing clusters of circulating tumor cells (CTCs) inside blood vessels. CTCs can also form cell clusters in lymphatic vessels, so called lymphatic vessel invasions (LVIs) (Rakha et al., 2012). However, they appear to lack TNC (Sun et al., 2019). BVIs vary in size and can occlude the vessel, a condition that can cause pulmonary embolism, a frequent cause of death in cancer patients (Sun et al., 2019; Fujii et al., 2014). In a murine breast cancer progression model, BVIs were found in the primary tumor and in the lung, and they had a similar organization in both locations (an outer endothelial layer, fibroblast layer and a CTC core), as seen in some human cancers, where TNC has been found to promote EC monolayer integrity (Sun et al., 2019) (Fig. 2B). The endothelial layer of the BVIs generated a continuum with the pulmonary endothelium, thereby likely promoting invasion of tumor cells into the lung parenchyma. Tumor cell entry into the lung was also promoted by TNC through epithelial-mesenchymal transition (EMT) involving platelets and transforming growth factor β (TGFβ) signaling (Sun et al., 2019). Moreover, the FBG domain of TNC has been recently shown to trigger TGFβR signaling through binding to the small latent TGFβ complex (SLC) including the latency-associated peptide (LAP), thus promoting TGFβ maturation (Aubert et al., 2021) (Fig. 1B).

How TNC impacts the function of lymphatic vessels in tumors is not clear. The abundance of lymphatic endothelial cells (LECs) was not obviously affected by the absence of TNC in several experimental murine tumors (Saupe et al., 2013; Spenlé et al., 2020; Murdamoothoo et al., 2021). However, TNC and endothelin-1 (ET-1, also known as EDN1) were identified as inducers of lymphatic vessels upon podoplanin overexpression in MCF7 breast tumor xenografts (Cueni et al., 2010). As TNC induced ET-1 and its receptor EDNRA in T98G GBM cells (Lange et al., 2007), as well as induced CCL21 in lymphatic endothelial cells (Spenlé et al., 2020), an impact of TNC on lymphatic vessel formation and functions in some cancers is likely and needs to be addressed in the future.

TNC has been shown to induce vasculogenic mimicry (VM), where invasive cancer cells form vessel-like structures by differentiation into TDECs (Cai et al., 2019; Kang et al., 2021). VM is considered to be responsible for the failure of anti-angiogenic therapy, because it promotes tumor aggressiveness, and for poor prognosis (Williamson et al., 2016). In gliomas, TNC decreased Akt phosphorylation, leading to downregulation of matrix metalloproteinase (MMP)2 and MMP9, and promoting VM (Cai et al., 2019). In a model of gastric cancer, TNC induced VM by promoting EMT. Importantly, TNC-KD inhibited VM (Kang et al., 2021). Thus, inhibition of TNC and downstream molecules that promote leakiness and VM might be useful for counteracting anti-angiogenic therapy resistance.

## Tenascin-C promotes plasticity, invasion and metastasis

It is well established that high levels of TNC expression correlates with increased invasion, more metastasis and often shorter patient survival (Midwood et al., 2016; Lowy and Oskarsson, 2015). Several murine models with engineered levels of TNC have been developed to investigate the functions of TNC in tumorigenesis and metastasis (Table 1). These studies revealed that TNC acts on many cells and employs several signaling pathways, including Wnt, Notch, TGFβ, TLR4 and others (reviewed in Orend and Chiquet-Ehrismann, 2006; Midwood et al., 2011, 2016) (Fig. 2C,D). TNC overexpression in the pancreatic islets of a PNET model, which mimics high TNC levels in tumors, caused increased lung micrometastasis (Saupe et al., 2013). Furthermore, overexpression of TNC (through CRISPR/Cas9 technology) confirmed a metastasis-driving role of TNC in a human lung carcinoma grafting model (Gocheva et al., 2017). The authors showed that guide RNA (gRNA)-induced high levels of TNC in tumor cells injected into in the tail vein caused increased lung metastasis (Gocheva et al., 2017) (Table S1).

### Role of extracellular vesicles

Recent literature suggests that tumor cells prepare the site for tumor cell homing (the so-called ‘pre-metastatic niche’) for future metastasis by employing several cell types, including fibroblasts, ECs, macrophages and likely others. Moreover, TNC plays an important role in this pre-metastatic niche (Hongu et al., 2022). With regard to how this niche is prepared, lactic acid-associated low pH (Riedel et al., 2022) (with no explicit link to TNC so far) and extracellular vesicles (EVs) secreted by the tumor have become a recent focus of interest (Guo et al., 2019; Dong et al., 2021). Carcinoma-associated fibroblasts (CAFs) have been shown to secrete EVs containing ECM components including TNC (Albacete-Albacete et al., 2020; Rai et al., 2019; Mirzaei et al., 2018). Although local deposition of TNC at the invasive front has been suggested to promote cancer cell invasion (Oskarsson et al., 2011), TNC deposition in the lung by EVs (caveolin-1 dependent) might facilitate the formation of a pulmonary pre-metastatic niche (Albacete-Albacete et al., 2020; Doglioni et al., 2019; Dong et al., 2021; Liu and Cao, 2016; Peinado et al., 2017). In urinary bladder carcinoma patients (without any detectable metastasis in the lymph nodes), TNC was largely increased in the apparently healthy lymph node tissue where it forms dense networks that are remarkably reminiscent of TNC tracks in primary tumors (Silvers et al., 2021). Moreover, high TNC expression in the lymph nodes correlated with shorter patient survival. The authors showed that EVs (isolated from urine) contained several factors that induce TNC expression in fibroblasts (through the NFκB pathway) (Silvers et al., 2021). Altogether, through tumor EVs, TNC can become either locally induced (lymph nodes) or be directly deposited (lung), potentially playing a role in the preparation of a metastasis-promoting microenvironment (Fig. 2D).

### Roles of mechanosignaling

A link between TNC and mechanosignaling has been reported in GBM where tissue stiffness was shown to correlate with malignancy and high TNC expression (Miroshnikova et al., 2016). The authors identified ECM stiffness as an inducer of hypoxia-inducible factor 1α (HIF1α) (which in turn induces TNC) and a repressor of miR203 (which downregulates TNC), thus enhancing stiffness and tumor malignancy. In support, TNC-KD reduced stiffness in a GBM xenograft model and prolonged survival of the tumor mice (Miroshnikova et al., 2016).

In fibroblasts and epithelial cells, the mechanosensor megakaryoblastic leukemia 1 (MKL1; also known as MRTFA) has been shown to induce TNC; this occurs in a serum response factor (SFR)-dependent and SFR-independent manner, requiring, in the latter case, the SAP domain of MKL1. SAP is a homology domain found in nuclear scaffold attachment factors SAF-A and -B, in the acinus protein, required for the condensation of apoptotic chromatin, and in PIAS. The SAP domian directly binds to DNA in the proximal part of the TNC promoter. SAP-dependent expression of TNC correlates with shorter survival of breast cancer patients (Asparuhova et al., 2011; Gurbuz et al., 2014). Vice versa, TNC can counteract (FN-induced) mechanosignaling by impairing the formation of focal adhesions and inactivating RhoA through inhibition of syndecan-4, an important co-receptor of α5β1 integrin, thus altogether likely alleviating high tension that damages the tissue (Huang et al., 2001; Midwood et al., 2006; Van Obberghen-Schilling et al., 2011).

The Hippo signaling pathway, which is mediated by proteins including YAP and transcriptional coactivator with PDZ-binding motif (TAZ; also known as WWTR1) proteins is another important mechanosignaling pathway. Translocation of YAP and TAZ (hereafter YAP/TAZ) into the nucleus allows their binding to members of the transcriptional enhanced associated domain (TEAD) family of transcription factors, thereby activating the expression of genes involved in cell adhesion and migration (Piccolo et al., 2014; Moroishi et al., 2015). TNC has been shown to inhibit YAP/TAZ signaling (Lee et al., 2022; Sun et al., 2018; San Martin et al., 2017). TNC caused cell rounding (impairing actin stress fiber formation) through integrin α9β1 (presumably by an autocrine mechanism), which impaired nuclear translocation of YAP, thus promoting amoeboid Boyden chamber transwell migration and lung metastasis (Sun et al., 2018). Downstream, several genes were repressed, and a lowered expression of these genes correlated with shorter survival of cancer patients when TNC levels were lower than the median (Table 1). Lower TNC levels are usually considered to indicate a better prognosis; however, these TNC levels can still be considered as largely elevated in comparison to normal tissues, which have negligible TNC expression. Inhibition of YAP mechanosignaling by TNC was also recently found in ankylosing spondylitis where TNC promoted entheseal new bone formation (Li et al., 2021). The authors generated a novel murine TNC-KO model with a CRISPR/Cas9-mediated KO (or applied an inhibitory TNC antibody) and found that although YAP-mediated mechanosignaling was inactive, TNC activated overall YAP/Hippo signaling, thereby increasing the expression of chondrogenic genes and subsequent bone formation (Li et al., 2021).

As YAP/TAZ signaling promotes cancer progression it is considered an anti-cancer target, and several YAP/TAZ-inhibiting drugs are currently under development, with some already employed in clinical trials (Pobbati and Hong, 2020). However, in LGG or in GBM [with the R132H mutation in isocitrate dehydrogenase 1 (IDH1) which correlates with better GBM patient survival (Miroshnikova et al., 2016)], targeting YAP might be cautioned as YAP inhibition might promote tumor cell dissemination and metastasis (Sun et al., 2018). Thus, TNC appears to have a cell- and context-dependent effect on YAP/TAZ and MKL1 signaling that should be taken into account for anti-cancer therapy. Moreover, as soon as it is expressed, TNC can drive pro-tumorigenic actions.

### Roles of integrin α5β1

In several studies, integrin α5β1 has been proposed to be involved in mediating responses towards TNC. TNC has been shown to inhibit T cell proliferation, which involved α5β1 present on T cells, and downregulated Akt and mammalian target of rapamycin (mTOR) signaling (Hauzenberger et al., 1999; Jachetti et al., 2015; Mirzaei et al., 2018). TNC has also been proposed to promote tumor progression by inducing integrin α5β1-mediated YAP activation and upregulating metastasis-associated lung adenocarcinoma transcript 1 (MALAT1) in Ewings sarcoma (He et al., 2019). Moreover, TNC has a cryptic interaction site in the FNIII domain A2 (TNIIIA2), which becomes exposed upon proteolytic cleavage by MMP2 or MMP9 (Saito et al., 2007). A peptide derived from TNIIIA2 has been shown to bind the ectodomain of syndecan-4, triggering α5β1 activation, which conferred anoikis resistance, PDGF-dependent cell proliferation and GBM cell survival, proliferation and migration (Saito et al., 2007; Tanaka et al., 2014; Iyoda et al., 2020). Although α5β1 integrin is involved in the described TNC responses, there is no experimental evidence that this RGD-dependent integrin is a receptor for TNC. Of note, human TNC has an RGD sequence in the TN3 domain, which is not the case for murine TNC. Alternatively, FN, the natural and so far only confirmed ligand of α5β1, is often co-expressed with TNC (Pankov and Yamada, 2002; Van Obberghen-Schilling et al., 2011). Thus, inhibition of α5β1 might block FN responses, enhancing the actions of TNC.

### EMT

There are several reports that describe that TNC plays a role in EMT (Sun et al., 2019; Lüönd et al., 2021; Saxena et al., 2021; Nagaharu et al., 2011; Katoh et al., 2013). Recently, by using a TNC-promoter-driven inducible Cre recombinase, EMT was detected *in vivo* in grafted tumor cells through it leading to removal of a stop sequence in a floxed fluorophore (Lüönd et al., 2021). This model could be highly valuable for providing information about the identity of cells and conditions when TNC is expressed during tumor onset and tumor progression (Table S1). The same group recently discovered that the lncRNA ET-20 is instrumental in EMT (Saxena et al., 2021). The authors reported that TGFβ signaling induced ET-20 through the transcription factor Sox4. Loss-of-function experiments showed that ET-20 regulates TNC protein levels and that the TNC protein regulated ET-20 expression, uncovering a yet incompletely understood crosstalk. Moreover, both ET-20 and TNC were required for TGFβ-induced EMT; here, ET-20 might destroy cell–cell junctions, as it was found to directly bind to desmosomal proteins (Saxena et al., 2021).

### Tenascin-C shapes tissue and tumor immunity

#### Roles of TNC in tissue immunity

TNC can play pro- and anti-inflammatory roles in injured tissues, which is context dependent as recently reviewed for myocarditis, osteoarthritis, rheumatoid arthritis, gliosis, systemic sclerosis and fibrosis (Bhattacharyya et al., 2022; Imanaka-Yoshida, 2021; Hasegawa et al., 2020; Marzeda and Midwood, 2018). Briefly, TNC can act as a DAMP, through engaging TLR4 and integrin α9β1 in signaling pathways through direct interaction of TNC with these cell surface receptors, leading to the induction of pro-inflammatory cytokines, such as TNF, IL6, IL8 and others. This has been noted in many types of immune cells [such as macrophages and dendritic cells (DCs)], as well as fibroblasts and chondrocytes (Marzeda and Midwood, 2018; Midwood et al., 2016; Imanaka-Yoshida, 2021). High TNC levels in serum correlated with the critically ill status of patients (Xu et al., 2021). TNC was also found to be highly expressed in bacterially infected tissues, and during sepsis (Meijer et al., 2020, 2021a,b). In addition, TNC was also found to be upregulated in the bronchoalveolar lavage of critically ill COVID-19 patients (Zeng et al., 2021). In a recently established mouse model with inducible overexpression of TNC in cardiomyocytes, the elevated TNC levels caused premature death accompanied by inflammation (Yonebayashi et al., 2021). However, surprisingly, the mice lacked a significant heart phenotype (Yonebayashi et al., 2021). Of note, this model with inducible TNC overexpression could be highly valuable for addressing the roles of high TNC in cancer progression (Table S1).

#### Impact of TNC on leukocytes

TNC can promote the recruitment, activation and polarization of T lymphocytes. TNC can also inhibit the proliferation and transmigration of T lymphocytes (reviewed in Imanaka-Yoshida, 2021). DCs are activated following stimulation by TNC indicated by increased expression of CD86, CD80 and secretion of mature IL1 (Machino-Ohtsuka et al., 2014; Spenlé et al., 2020). DCs isolated from TNC-KO mice showed an attenuated cellular response to LPS with reduced pro-inflammatory cytokine secretion (Ruhmann et al., 2012). Furthermore, a profound influence of TNC on the polarization and activation of macrophages has also been demonstrated (Liu et al., 2012; Piccinini et al., 2017). This also applies to microglial cells (resident macrophages of the central nervous system) where TNC, through activation of TLR4 signaling and a not well understood involvement of histone deacetylase 1 (HDAC1), upregulated expression of IL6 and TNF (Haage et al., 2019). In addition to activating TLR4, TNC is able to bind to α9β1 and αvβ3 integrins, which leads to an increase in pro-inflammatory cytokines (e.g. IL6 and CCL2) (Kanayama et al., 2009). The many actions of TNC in inflammation and infection suggest multiple and important roles in immunity control and raise the question of whether TNC has co-evolved with factors that are important for immunity. Indeed, TNC evolved in jawed fish at the time when the CXC and CC chemokines and IgG adaptive immunity also appeared (Orend and Tucker, 2021).

### TNC regulates immune suppression

**Fig. 3.**
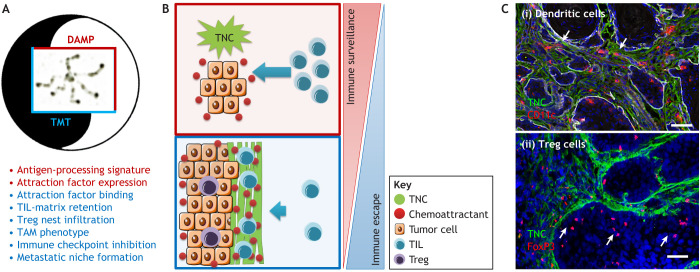
**TNC regulates tumor immunity.** (A) TNC can have opposing effects on tumor immunity. TNC might act as a DAMP by upregulating genes involved in antigen processing and presentation, but it also promotes Treg infiltration, upregulation of immune checkpoint molecules and the formation of tumor matrix tracks (TMTs) that immobilize a subset of TILs in the stroma. (B) As soon as TNC is expressed, TILs might be attracted into the tumor by factors expressed by the tumor cells (such as CXCL12), thus supporting immune surveillance (top). However, as soon as TMTs are formed, the immune surveillance and DAMP function of TNC is overcome by TIL retention, thus blocking access to the tumor cells (bottom). (C) Representative images of dendritic cells (CD11c, red) (i), and Treg (FoxP3, red) (ii) from 4NQO tongue OSCC. Reproduced from Spenlé et al. (2020) under rights retained by G.O. Fig. 3C is not published under the terms of the CC-BY license of this article. For permission to reuse, please see individual reference. Scale bars: 50 µm. The white arrows point at TILs retained in the stroma (I) or attracted into the tumor nest (II).

An enrichment of leukocytes (in particular T cells) in areas with abundant TNC has been observed in human lung cancer and human GBM tissue (Onion et al., 2018; Huang et al., 2010), as well as in murine models of GBM (Huang et al., 2010), breast cancer (Talts et al., 1999), PNET (Spenlé et al., 2015a) and prostate cancer (Jachetti et al., 2015). These observations are suggestive of a potential impact of TNC on tumor immunity; however, just how TNC acts remained largely unknown until recently. In many tumors TNC forms matrix niches organized as tracks, so called tumor matrix tracks (TMTs) (Spenlé et al., 2015a; Tomko et al., 2018) that exert immunoregulatory functions (Fig. 2A–D). In tumor models with stochastic tumor onset and engineered TNC levels, TNC was found to orchestrate tumor immunity. In tongue oral squamous cell carcinoma (OSCC) (Spenlé et al., 2020) and the MMTV-NeuNT and NT193 breast cancer progression models (Murdamoothoo et al., 2021), TNC upregulated and bound the chemokines CCL21 and CXCL12, respectively, thereby attracting and retaining tumor-infiltrating leukocytes (TILs) in the stroma. This affected DCs in the OSCC model, and the function of macrophages and CD8T cells in the MMTV-NeuNT and NT193 breast cancer models (Spenlé et al., 2020; Murdamoothoo et al., 2021). Inhibition of CCR7 (the receptor for CCL21) and CXCR4 (the receptor for CXCL12), respectively, released DC and CD8T cells from TNC, thus identifying ‘TIL-matrix retention’ as a novel mechanism for how TNC impairs anti-tumor immune surveillance, and also provided an explanation for the immune exclusion phenotype where TILs are found to be trapped in the stroma, an indicator of poor efficacy of immune checkpoint therapy (Petitprez et al., 2020; Pagès et al., 2018). TNC further promoted infiltration of regulatory T cells (Treg), which was abolished upon CCR7 inhibition (OSCC model) (Spenlé et al., 2020) (Fig. 3). TIL-matrix retention is also relevant in human head and neck squamous cell carcinomas (HNSCCs) and in human breast cancer patients (Table 1).

It has also been suggested that B16 melanomas and other tumors might mimic the immunosuppressive properties of lymph nodes, as these tumors show numerous CCR7+ cells and high CCL21 expression (Shields et al., 2010). This intriguing possibility seems to apply also to HNSCCs, where in addition to high CCL21 and numerous CCR7+ cells, lymph node fibroblastic reticular cells (FRCs) were also found to be highly abundant inside the matrix tracks (Spenlé et al., 2020). Importantly, TNC promotes lymphoid immune suppression, which is largely abolished in TNC-KO tumors (Spenlé et al., 2020).

As the TNC-KO mouse is viable (Table S1), this mouse model has proven a useful tool to interrogate whether host- and tumor-derived TNC have similar or distinct functions. To that end, tumor cells with a TNC-KD were grafted into either the wild-type (WT) or TNC-KO host (Deligne et al., 2020). A close inspection of the macrophage phenotype revealed that TNC expressed by the host directed macrophages towards an M1 phenotype (TNC likely acting as DAMP). In contrast, tumor cell-derived TNC forced macrophages towards a M2-like tumor-associated macrophages (TAM) phenotype, thereby promoting tumor growth and lung metastasis (Deligne et al., 2020). In gliomas, KO of CD47 induced TNC, which promoted phagocytosis and revealed a poorly understood immune crosstalk that is affected by TNC (Ma et al., 2019). Investigating gene expression patterns revealed that tumors with host-derived TNC upregulated several genes associated with antigen processing and presentation, shedding light on a potential, but as yet poorly understood, DAMP function of TNC in cancer (Deligne et al., 2020; Spenlé et al., 2021; Murdamoothoo et al., 2021). The balance between pro- and anti-tumorigenic effects of TNC on immune surveillance might regulate whether a tumor thrives or regresses (Fig. 3A,B). It is interesting to note that normal and activated fibroblasts, monocyte-derived dendritic cells (MDDCs) and T cells all express TNC splice forms that differ from that of resting fibroblasts and tumor cells, which affect immune functions of MDDCs such as expression of cytokines (e.g. IL6 and TNFα) (Giblin et al., 2020). Interactions of MDCCs with the AD2 and AD1 domains of TNC kept them in a roaming state, whereas interactions with TNC variants lacking these domains caused their activation (Giblin et al., 2020).

#### TNC-mediated macrophage–EC crosstalk

Transcriptomic analysis of ECs from murine lung metastasis combined with loss- and gain-of-function studies has revealed that cancer-cell-derived TNC activates secretion of TNFα and nitric oxide in macrophages in a TLR4-dependent manner (Hongu et al., 2022). This triggers gene expression in ECs that promoted metastasis where expression of four factors, INHBB (inhibin β B), laminin α1, osteoprotegerin (OPG) and secreto-globulin family 3A member 1), were found to be crucial and correlated with worsened survival of breast cancer patients (Hongu et al., 2022) (Fig. 2D). Furthermore, staining of lung metastasis revealed a track-like organisation of TNC and laminin (Sun et al., 2019; Murdamoothoo et al., 2021), similar to that seen in the primary tumor accompanied by an accumulation of leukocytes (in particular CD8+ T cells) in the matrix (Murdamoothoo et al., 2021) (Fig. 2A,B). Thus, a similar mechanism might underlie the formation of a stromal niche in the primary tumor and in the metastatic lung, with TNC as a common determinator (Fig. 2D).

### Targeting TNC

Targeting the tumor matrix has recently got some attention (Cox, 2021). Given its prominent expression in cancer and multiple pro-tumorigenic roles, TNC might represent a valid anti-cancer target (reviewed in Spenlé et al., 2015b; Tucker and Degen, 2022). Briefly, antibodies, nanobodies and novel peptides are being used to improve radiotherapy or drug uptake into tumor tissues, as well as for tumor imaging (reviewed in Tucker and Degen, 2022). Similarly, repression of TNC expression by siRNA and aptamers has also been exploited (reviewed in Spenlé et al., 2015b). Drug-induced stress signaling as measured by pan JNK activation has been shown to induce TNC and osteopontin (OPN), counteracting its potential as anti-cancer treatment, but inhibition of JNK (thus downregulating TNC and OPN) could sensitize breast cancer patients to chemotherapy (Insua-Rodriguez et al., 2018). Moreover, TNC is induced in PyMT tumors by a subset of CAFs expressing integrin α11β1. The interaction of the integrin α11β1 with PDGFRβ in a PDGF-ligand-dependent manner leads to activation of the JNK pathway, which had been previously shown to induce TNC (Mettouchi et al., 1997). In this context, the combined inhibition of JNK and PDGFRβ was able to reduce metastasis (Primac et al., 2019), offering additional opportunities to control TNC expression. Recently, TNC abundance was shown to be regulated by autophagy (Li et al., 2021). As autophagy is impaired in triple-negative breast cancer, the authors conclude that elevated TNC levels in this cancer subtype might be due to impaired TNC degradation (Li et al., 2021). It will be interesting to see whether impaired autophagy also has an impact on TNC expression in fibroblasts, the major source of TNC in breast cancer.

Targeting pathways that are activated by TNC represents an additional approach. A recurrent pattern is apparent in that TNC enforces the regulation of a pathway at several levels, with the following non-exhaustive examples. First, TGFβ signaling, where TGFβ induces TNC and lncRNA ET-20, both regulating expression of each other and being important for TGFβ-induced EMT (Saxena et al., 2021). TGFβ binds to TNC (TN3–TN5 domains), thereby sequestering TGFβ in the matrix (De Laporte et al., 2013; Loustau et al., 2022). The FBG domain of TNC binds to LAP and activates latent TGFβ, inducing subsequent signaling (Aubert et al., 2021). Second, Wnt signaling, where, in cultured tumor cells, TNC binds to the Wnt3a ligand, thus either promoting (when TNC and Wnt3a are offered as substratum) or inhibiting Frz signaling (when offered as soluble molecules) (Hendaoui et al., 2014). TNC also downregulates the Wnt pathway repressor DKK1 (through inhibition of integrin α9β1 and downstream YAP), thus likely further enforcing Wnt/Frz signaling (Saupe et al., 2013; Sun et al., 2018). Third, CCR7 signaling, where TNC increases the abundance of FRCs in OSCC, thus providing a source of CCL21. Moreover, TNC induces CCL21 in LECs (through α9β1 integrin), as well as its receptor CCR7 (through TLR4) on several types of leukocytes, thereby likely promoting their activation and entry into the tumor through lymphatic vessels (Spenlé et al., 2020). TNC binds to CCL21 via TN3–TN5 domains, thereby retaining CCR7+ leukocytes in the stroma and impairing CD11c+ cell functions. Finally, CXCR4 signaling, where TNC induces CXCL12 in tumor cells (through TLR4), but also binds to CXCL12 (again in the TN3–TN5 domains) thus trapping CD8T cells in the stroma, exerting a Janus function (i.e. both activation and inhibition) on yet another pathway (Murdamoothoo et al., 2021). Taken together, the multiple levels by which TNC regulates these pathways might reflect the importance of TNC in normal tissue and immune homeostasis, which becomes out of control in cancer.

**Fig. 4.**
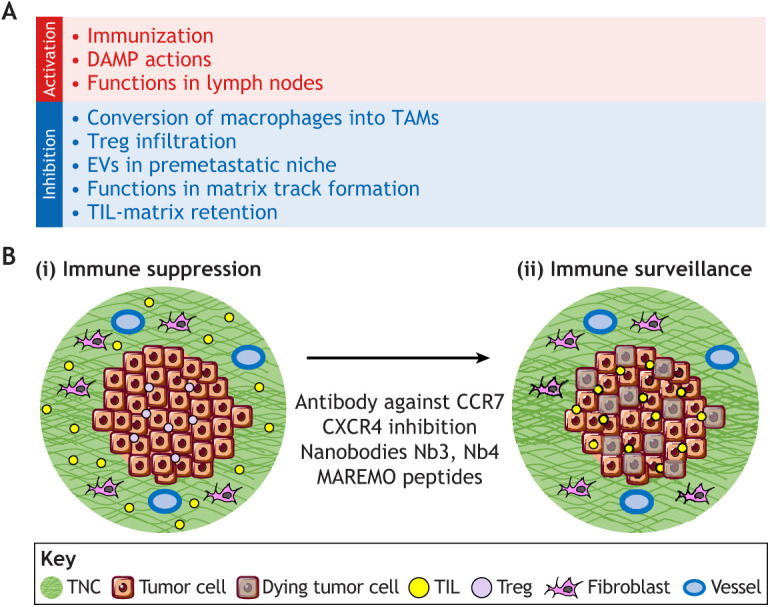
**Targeting the immune functions of tenascin-C in cancer.** (A) Summarized here are approaches for targeting the diverse functions of TNC in immunity that could be used to improve the anti-tumor immune surveillance phenotype. (B) Targeting ‘TIL-matrix retention’ represents a novel opportunity to target TNC in cancer, potentially abolishing the immune-exclusion phenotype and empowering immune checkpoint therapies that do not require the lowering of TNC expression, offering the opportunity to potentially preserve its desirable DAMP actions.

To finetune immune responses and avoid autoimmunity, the body employs several feedback mechanisms, including inhibition of CD8T cells through so called immune checkpoints, which are often found to be activated in tumors, offering a window for therapeutic intervention (Sharma and Allison, 2020). However, these so called immune checkpoint therapies are found to be confounded by the immune-exclusion phenotype, which is characterized by T cells being trapped in the (TNC-rich) stroma, likely explaining poor efficiency of this approach (Zemek et al., 2020; Galon and Bruni, 2019; Fares et al., 2019) (Fig. 3). Thus, targeting the matrix, and in particular TNC and its immune suppressive actions, could be an attractive novel opportunity to restore TIL cytotoxicity (Fig. 4A,B). Inhibition of CXCR4 and CCR7 phenocopied TNC-KO tumors, in which the expression of immune-suppressive factors (e.g. PDL-1, PD-1, CTLA4, TGFβ and IL10) was largely reduced, thus revealing TNC as an important regulator (Spenlé et al., 2020; Murdamoothoo et al., 2021). Also, CXCR4 inhibition in combination with anti-CTLA4 treatment was superior over each treatment alone in reducing tumor growth in a pancreatic tumor model (Feig et al., 2013). Similarly, targeting TNC in a breast cancer progression model together with anti-PDL-1 treatment was also more potent in reducing metastasis than either treatment alone (Deligne et al., 2020). It was also noted that TNC promoted Treg infiltration; however, how is unknown. Thus, targeting these diverse functions of TNC acting as DAMP, activating Treg, and retaining TIL in the stroma might boost the body's own anti-cancer immunity (Fig. 4A). It is likely that TNC impacts even more immune subtypes, which needs to be better understood for its exploitation as an anti-cancer treatment.

Recently, novel TNC-targeting tools were developed that abolish the recently described ‘TIL-matrix retention’ function of TNC by releasing TILs from the stroma. These comprise TNC-specific nanobodies (Nb3 and Nb4) (Dhaouadi et al., 2021) and so-called matrix regulating motif (MAREMO)-containing peptides (Loustau et al., 2022). Interestingly, the TNC-specific nanobodies associated with the central TN3–TN5 domains of TNC (Dhaouadi et al., 2021), where the two MAREMO peptides were also found to bind (Loustau et al., 2022). These central FNIII domains (TN3–TN5) of TNC appear to interact with several factors, including FN (Loustau et al., 2022), TGFβ and other soluble factors (De Laporte et al., 2013), as well as with integrins (e.g. αvβ3 and α9β1) (Midwood et al., 2016) and antibodies from camel (Dhaouadi et al., 2021) (Fig. 1B). Interestingly, both these nanobodies and peptides released cultured DCs from a TNC–CCL21 substratum, opening novel opportunities for potential future *in vivo* applications (Dhaouadi et al., 2021; Loustau et al., 2022) (Fig. 4B). Remarkably, the MAREMO peptides not only abolished the TIL-matrix retention function of TNC, but also blocked its interaction with FN and inhibited TGFβ signaling, as well as FN and collagen matrix expression and subsequent matrix assembly. Thus, the MAREMO peptides might affect several functions of TNC, in particular causing reduction of matrix density, which could be relevant for cancer and fibrosis (Loustau et al., 2022).

How could we use our knowledge for a tailored anti-cancer therapy? Expression of TNC in the tumor tissue as well as in blood could be used for detection of tumors and CTCs, respectively. Activating a moderate DAMP function of TNC together with immunization using established protocols that include TNC as a tumor antigen (reviewed in Spenlé et al., 2015b) could help the body to launch an anti-tumor response. Blocking the immune-suppressive functions of TNC by abolishing TIL-matrix retention, enforcing TIL activation, inhibiting Treg functions and blocking the assembly of TNC into matrix tracks, in particular in combination with additional chemo-, radio- or immune-checkpoint therapies, could improve tumor regression and abolish metastasis. Finally, inhibiting EV-mediated deposition of TNC in premetastatic niches is also desirable (Fig. 4A,B). Ultimately, we should aim for a tissue normalization after anti-cancer targeting in order to avoid future tumor relapse, and, here, the poorly understood normal functions of TNC in healthy tissues (e.g. in glomeruli, the gastrointestinal tract and lymph nodes) have to be respected.

## Conclusions and outlook

With the recent advent of novel tumor models, comprehensive omics and pathway analysis, as well as novel targeting tools, we have gained significant insight into TNC as a critical regulator of tumor immunity. Although TNC is more famous for enhancing pathological conditions, TNC also has multiple, although incompletely understood functions, in normal tissue homeostasis, and also here, an important role in immunity control is emerging. A better understanding of the physiological functions of TNC could guide the development of novel approaches to a tailored targeting of TNC in cancer. Moreover, as different TNC proteoforms exist, the next challenge will be to attribute distinct functions to specific TNC proteoforms, in particular during tumor–immune system evolution and in tumors that escape anti-cancer therapies. During this quest, immune-competent tumor models with a human tumor-like tumor microenvironment are especially promising means of providing important information about TNC for future anti-cancer therapies in the human patient.

## Supplementary Material

10.1242/joces.260244_sup1 Supplementary information
