# Supplementary material for: Advances on the roles of tenascin-C in cancer
Source: J Cell Sci. 2022 Sep 14;135(18):jcs260244. doi: 10.1242/jcs.260244 (PMC9584351; doi:10.1242/jcs.260244)
Supplement: Supplementary information [file joces-135-260244-s1.pdf]

Table S1. TNC engineered mouse models amenable for cancer research

Mouse	Reference
**TNCKO mouse**	
Insertion of LacZ gene into exon 2 [(a)]<br>Deletion of exon 2 [(a)]<br>Deletion of exon 2 [(a)]<br>CRISPR/Cas9 deletion of exon 3-5 [(b)]	Saga et al., 1992 [(1)]<br>Forsberg et al., 1996 [(2)]<br>Evers et al., 2002 [(3)]<br>Li et al., 2021 [(4)]
MMTV-NeuNT mammary gland tumors and lung metastasis in TNCKO mouse [(2, c)]	Murdamoothoo et al., 2021 [(2)]<br>Sun et al., 2019 [(2)]
4NQO tongue OSCC and lymph node metastasis in TNCKO mouse [(2, b)]	Spenle et al., 2020 [(2)]
Stochastic rat insulin promoter-driven SV40-induced PNET in TNCKO mouse [(b)]	Langlois et al., 2014 [(2)]<br>Saupe et al., 2013 [(2)]
*m*4T1 (mammary gland) [(d)] and *m*CT26 cells (colorectal carcinoma cells injected into the spleen) [(b)] grafted tumors in TNCKO mouse [(2)]	O`Connell et al., 2011 [(2)]
Stochastic PyMT mammary gland tumors and lung metastasis in TNCKO mouse [(2, a)]	Talts et al., 1998 [(2)]
**TNCKD cells (shTNC) / immune competent host**	
*m*4T1 (mammary gland) [(d)] and *m*EO771 (mammary gland) [(b)] grafted tumors and lung metastasis in WT mouse	Hongu et al., 2022
*m*4T1 cell grafted mammary gland tumors in WT mouse [(d)]	Li et al., 2021
*m*NT193 cell grafted mammary gland tumors and lung metastasis in TNCKO mouse [(c)]	Murdamoothoo et al., 2021 [(2)]<br>Deligne et al.. 2020 [(2)]<br>Sun et al., 2019 [(2)]
*m*AGR53 (Hras (Harvey Rat sarcoma virus)/shp53 transformed) primary astrocytes grafted brain tumors in WT mouse [(b)]	Angel et al., 2020
*m*TPIN-SC cell grafted prostate/subcutaneous tumors in WT mouse [(b)]	Jachetti et al., 2015
**TNCKD cells (shTNC) / immune compromised host** (see Table 1)	
*h*MDA MB231-LM2 cell grafted mammary gland tumors and lung metastasis in nude mouse [(e)]	Hongu et al., 2022
*h*MDA MB231 cell grafted mammary gland tumors in nude mouse [(e)]	Li et al., 2021
*h*U87MG cells grafted subcutaneous tumors in nude mouse [(e)]	Rupp et al., 2016
*h*SW480 cells (WT) engrafted into RAG2KO or RAG2/TNCKO mouse [(f, 2)]	Spenlé et al., 2015
**TNC overexpressing mouse**	
Nkx2.5 promoter driven *m*TNC overexpression in cardiomyocytes [(b)]	Yonebayashi et al., 2021
CRISPR/Cas9 *m*TNC overexpression in *m*KP (KrasG12D/Trp53 null) lung carcinoma cells subcutaneous grafted tumors/lung metastasis in WT mouse [(b)]	Gocheva et al., 2017
Rat insulin promoter-driven *h*TNC over expression in Rip1Tag2 PNET [(b)]	Saupe et al., 2013 [(2)]
**TNC indicator mouse**	
*m*TNC promoter-driven Cre-recombinase expression to induce floxed eGFP [(c)]	Lüönd et al., 2021

Mouse models with engineered TNC levels have been generated either with a KO or an overexpression of TNC. The TNCKO mouse was generated four times independently targeting exon 2 (Saga et al., 1992; Forsberg et al., 1996; Evers et al.2002) or exon 3-5 (Li et al., 2021). Mostly the “Forsberg” mouse was used for tumor studies listed. TNCKD tumor cells (accomplished by shRNA technonolgy) were engrafted into WT or immune compromised mice. A gain of function mouse was accomplished by overexpression of the human TNC coding sequence (lacking AD1, AD2) under control of the rat insulin promoter (Saupe et al., 2013) or by generating a compound mouse expressing the complete murine TNC coding sequence with floxed stop sequences that got removed by the Nkx2.5 promoter driven Cre-recombinase in myocytes (Yonebayashi et al., 2021). The inducible conditional TNC overexpression mouse is suitable for tissue or cell type specific TNC overexpression upon crossing with an appropriate Cre-recombinase expressing mouse. The TNC-promoter driven Cre-recombinase expressing mouse (TNC-reporter mouse) is suitable to monitor TNC expression during tumorigenesis. The floxed stop sequence of eGFP gets removed in cells expressing TNC (Lüönd et al., 2021). *h*TNC, human TNC, *m*TNC, murine TNC. (a) 129/sv, (b) C57Bl6, (c) FVB, (d) BalbC, (e) nude, (f) RAG2KO mouse. Saga et al., 1992 (1), Forsberg et al., 1996 (2), Evers et al., 2002 (3), Li et al., 2021 (4).

References

Angel, I., Kerman, O.P., Rousso-Noori, L., Friedmann-Morvinski, D. (2020) Tenascin C promotes cancer cell plasticity in mesenchymal glioblastoma, *Oncogene*, 39(46): 1-15.

Deligne, C., Murdamoothoo, D., Gammage, A.N., Gschwandtner, M., Erne, W., Loustau, T., Marzeda, A.M, Carapito, R., Paul, N., Velazquez-Quesada, I., *et al.* (2020) Matrix-targeting immunotherapy controls tumor growth and spread by switching macrophage phenotype, *Cancer Immunol Res*, 8(3): 368-382.

Evers, M.R., Salmen, B., Bukalo, O., Rollenhagen, A., Bösl, M.R., Morellini, F., Bartsch, U., Dityatev, A., Schachner, M. (2002) Impairment of L-type Ca^{2+} Channel-Dependent Forms of Hippocampal Synaptic Plasticity in Mice Deficient in the Extracellular Matrix Glycoprotein Tenascin-C, *The Journal of Neuroscience,* 22(16): 7177-7194.

Forsberg, E., Hirsch, E., Fröhlich, L., Meyer, M., Ekblom, P., Aszodi, A., Werner, S., Fässler, R., *et al.* (1996) Skin wounds and severed nerves heal normally in mice lacking tenascin-C., *Proceedings of the National Academy of Sciences*, 93(13): 6594–6599.

Gocheva, V., Naba, A., Bhutkar, A., Guardia, T., Miller, K.M., Li, C.M.-C., Dayton, T.L., Sanchez-Rivera, F.J., Kim-Kiselak, C., Jailkhani, N., *et al.* (2017) Quantitative proteomics identify Tenascin-C as a promoter of lung cancer progression and contributor to a signature prognostic of patient survival, *Proceedings of the National Academy of Sciences*, 114(28): 5625-5634.

Hongu, T., Pein, M., Insua-Rodríguez, J., Gutjahr, E., Mattavelli, G., Meier, J., Decker, K., Descot, A., Bozza, M., Harbottle, R., *et al.* (2022) Perivascular tenascin C triggers sequential activation of macrophages and endothelial cells to generate a pro-metastatic vascular niche in the lungs, *Nature Cancer*, 3(4): 486–504.

Jachetti, E., Caputo, S., Mazzoleni, S., Brambillasca, C.S., Parigi, S.M., Grioni, M., Piras, I.S., Restuccia, U., Calcinotto, A., Freschi, M *et al.* (2015) Tenascin-C Protects Cancer Stem–like Cells from Immune Surveillance by Arresting T-cell Activation, *Cancer Research*, 75(10): 2095–2108.

Journal of Cell Science • Supplementary information

Langlois, B., Saupe, F., Rupp, T., Arnold, C., van der Heyden, M., Orend, G., Hussenet, T. (2014) AngioMatrix, a signature of the tumor angiogenic switch-specific matrisome, correlates with poor prognosis for glioma and colorectal cancer patients, *Oncotarget*, 5(21): 10529-10545.

Li, Z., Chen, S., Cui, H., Li, X., Chen, D., Hao, W., Wang, J., Li, Z., Zheng, Z., Zhang, Z. (2021) Tenascin-C-mediated suppression of extracellular matrix adhesion force promotes entheseal new bone formation through activation of Hippo signalling in ankylosing spondylitis, *Annals of the Rheumatic Diseases*, 80(7): 891–902.

Lüönd, F., Sugiyama, N., Bill, R., Bornes, L., Hager, C., Tang, F., Santacroce, N., Beisel, C., Ivanek, R., Bürglin, T. (2021) Distinct contributions of partial and full EMT to breast cancer malignancy, *Dev Cell*, 56: 3203-3221.

Murdamoothoo, D., Sun, Z., Yilmaz, A., Riegel, G., Abou-Faycal, C., Deligne, C., Velazquez-Quesada, I., Erne, W., Nascimento, M., Mörgelin, M., *et al.* (2021) Tenascin-C immobilizes infiltrating T lymphocytes through CXCL12 promoting breast cancer progression, *EMBO Molecular Medicine*, 13:e13270.

O'Connell, J.T., Sugimoto, H., Cooke, V.G., MacDonald, B.A., Mehta, A.I., LeBleu, V.S., Dewar, R., Rocha, R.M., Brentani, R.R., Resnick, M.B., *et al.* (2011) VEGF-A and Tenascin-C produced by S100A4 $^{+}$ stromal cells are important for metastatic colonization, *Proceedings of the National Academy of Sciences*, 108(38): 16002–16007.

Rupp, T., Langlois, B., Koczorowska, M.M., Radwanska, A., Sun, Z., Hussenet, T., Lefebvre, O., Murdamoothoo, D., Arnold, C., Klein, A., *et al.* (2016) Tenascin-C Orchestrates Glioblastoma Angiogenesis by Modulation of Pro- and Anti-angiogenic Signaling, *Cell Reports*, 17(10): 2607–2619.

Saga, Y., Yagi, T., Ikawa, Y., Sakakura, T., Aizawa, S. (1992) Mice develop normally without tenascin., *Genes & Development*, 6(10): 1821–1831.

Saupe, F., Schwenzer, A., Jia, Y., Gasser, I., Spenlé, C., Langlois, B., Kammerer, M., Lefebvre, O., Hlushchuk, R., Rupp, T., *et al.* (2013) Tenascin-C Downregulates Wnt Inhibitor Dickkopf-1, Promoting Tumorigenesis in a Neuroendocrine Tumor Model, *Cell Rep,* 5: 482-492.

Spenlé., C., Loustau, T., Murdamoothoo, D., Erne, W., Divonne, S.B.-D.L.F., Veber, R., Petti, L., Bourdely, P., Mörgelin, M., Brauchle. E.-M., et al., (2020) Tenascin-c orchestrates an immune-suppressive tumor microenvironment in oral squamous cell carcinoma, *Cancer Immunology Research,* 8: 1122-38.

Sun, Z., Velázquez-Quesada, I., Murdamoothoo, D., Ahowesso, C., Yilmaz, A., Spenlé, C., Averous, G., Erne, W., Oberndorfer, F., Oszwald, A. (2019) Tenascin-C increases lung metastasis by impacting blood vessel invasions, *Matrix Biol,* 83: 26-47.

Talts, J.F., Wirl, G., Dictor, M., Muller, W.J., Fässler, R. (1999) Tenascin-C modulates tumor stroma and monocyte/macrophage recruitment but not tumor growth or metastasis in a mouse strain with spontaneous mammary cancer, J Cell Sci, 112: 1855-1864.

Yonebayashi, S., Tajiri, K., Hara, M., Saito, H., Suzuki, N., Sakai, S., Kimura, T., Sato, A., Sekimoto, A., Fujita, S., *et al.* (2021) Generation of Transgenic Mice that Conditionally Overexpress Tenascin-C, *Frontiers in Immunology*, 12: 620541.
